# Endovascular repair of symptomatic abdominal aortic aneurysm: a seminal case in West Africa

**DOI:** 10.4314/gmj.v55i4.13

**Published:** 2021-12

**Authors:** Lily Wu, Benard O Botwe

**Affiliations:** 1 Department of Surgery, University of Ghana Medical School, College of Health Sciences, Korle Bu, Accra; 2 Department of Radiography, School of Biomedical & Allied Sciences, CHS, University of Ghana, Korle Bu, Accra

**Keywords:** Symptomatic AAA, EVAR, Open repair, Ghana, West Africa

## Abstract

Abdominal aortic aneurysm (AAA) is a fatal disease with high perioperative morbidity and mortality. Endovascular AAA repair (EVAR) is associated with remarkable improvement in the morbidity, mortality and length of hospital stay relative to open operative repair. We report a 79-year-old man with epigastric pain, which was diagnosed to be due to AAA on a computerised tomography angiogram (CTA). His only risk factor was hypertension. He had endovascular repair in 2018, the first-ever in Ghana and West Africa. 2021 is the 3rd year of surveillance post- EVAR with no disease progression or complication. This seminal case is a beacon of hope in Ghana's resource-constrained healthcare system.

**Funding:**

None declared

## Introduction

In low- and middle-income countries like Ghana, data on Abdominal Aortic Aneurysm (AAA) is either scanty or non-existent. In addition to the above fact is the lack of endovascular repair (EVAR) of AAA in Ghana and West Africa until 2017. Possible reasons for this may be, lack of awareness of EVAR, lack of trained vascular surgeon and endovascular interventionalist, unavailability of infrastructure and equipment for EVAR, non-existence of comprehensive health policies concerning modern trends of surgical treatment and general lack or poor patronage of health insurance financing for health care and in particular EVAR, and the misconception that EVAR is expensive and not cost-effective.

However, epidemiological data indicate a rising trend in the prevalence of AAA in developing countries.^1^–^3^ The increasing trend of cases, lack of screening and surveillance systems to promptly identify AAAs,^4^–^7^, coupled with the absence of endovascular aortic aneurysm repair specialists in Ghana over the years, means that many people probably die of the condition without any accurate, timely diagnosis or proper treatment. Relative to open surgical repair, EVAR is associated with a significantly lower peri-operative mortality.^7^

EVAR is now performed in Ghana. We share our experience of a successfully performed first case of EVAR to manage symptomatic AAA in Ghana. The importance and relevance of this case report is that it gives a beacon of hope in terms of the availability of EVAR in Ghana, a globally accepted and relatively safe surgical treatment for a fatal disease, AAA.

## Case Report

### Patient History

A 79-year-old male reported to the vascular clinic of Korle Bu Teaching Hospital in August 2018 with severe epigastric pain, worsening over 4 months. He was known with hypertension well controlled on single anti-hypertension medication, amlodipine 10mg daily. He had no history of cigarette smoking, hypercholesterolemia or diabetes. He also had no history of dyspepsia suggestive of peptic ulcer disease. He had no known cerebral, cardiac or renal disease. He had an emergency appendicectomy 20 years before the presentation.

### Physical Examination

Physical examination revealed a tall elderly male with no evidence of chronic disease. Blood pressure was 130/80 mmHg, and pulse rate was 78 beats/min. A tender pulsatile mass (about 8cm in its widest diameter) was palpable in the epigastrium. He had full complement of peripheral pulses in both lower limbs, with no stigmata of chronic limb-threatening ischaemia (CLTI). A diagnosis of symptomatic AAA was made.

### Investigations

**Figure 1:**
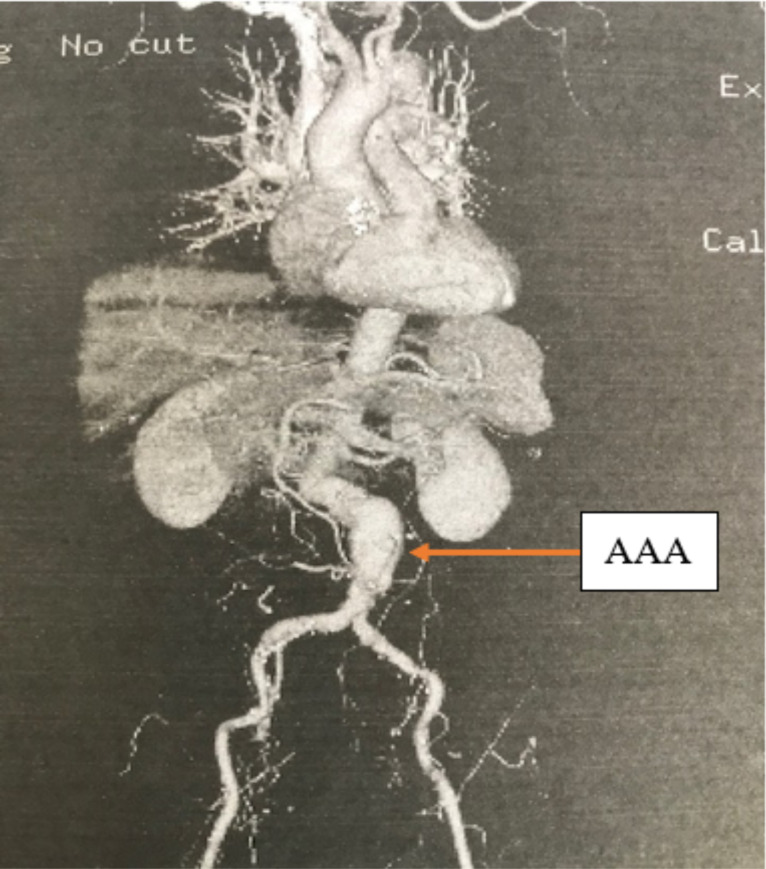
Pre-operative CT Angiogram

Several laboratory and radiological tests were performed. The various tests and the corresponding findings are presented in Table 1. A 3-dimensional (3D) reconstruction of the abdominal aortic aneurysm on a pre-operative computerised tomography angiogram (CTA) is presented in Figure 1.

**Table 1:** Laboratory and radiological examinations performed and their corresponding findings

Tests	Findings
**FBC**	HB 12.1g/dL(10–14) WBC 5.5 X 10^9^ (4–10) PLT 242x 10^9^ (150–400)
**BUE, Cr**	CR 72.0 (50–80) UREA 5.9 (2–7) NA 140 (135–145) K 4.2 (3.5–5.5)
**Chest x-ray**	Normal
**ECG**	Normal
**Stress ECG**	normal
**Computerised tomography** **angiogram (CTA)**	AAA diameter is 5.8 cm

### Management

The patient was started on tab clopidogrel 75 mg daily, tab atorvastatin 20 mg nocte and tab paracetamol 1g three times a day. He was clinically evaluated by the anaesthetist and declared fit for surgical repair.

**Figure 2:**
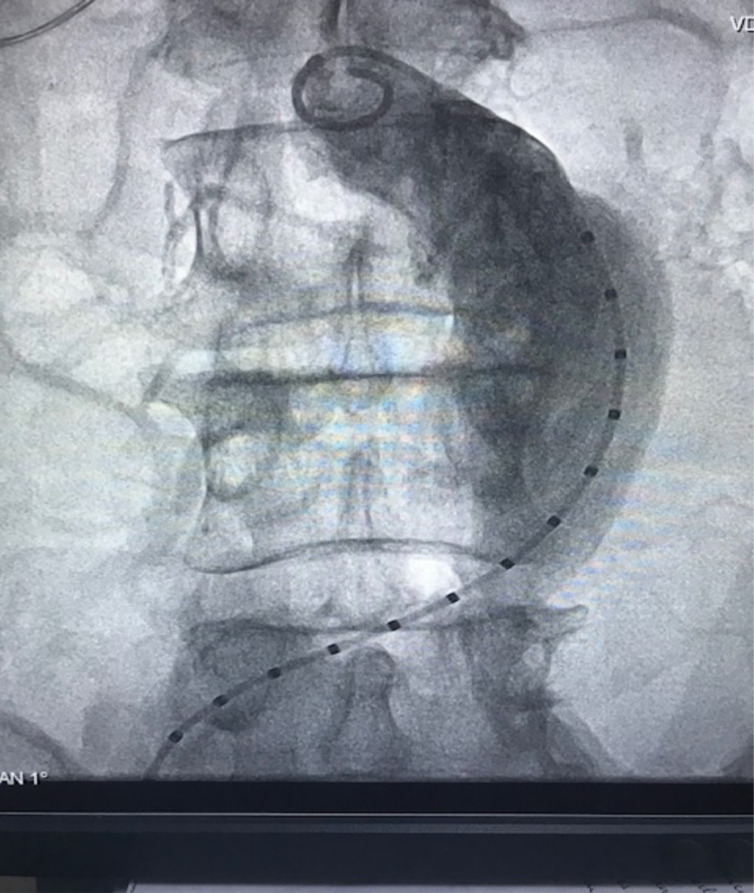
Intra-operative measurement of AAA

**Figure 3:**
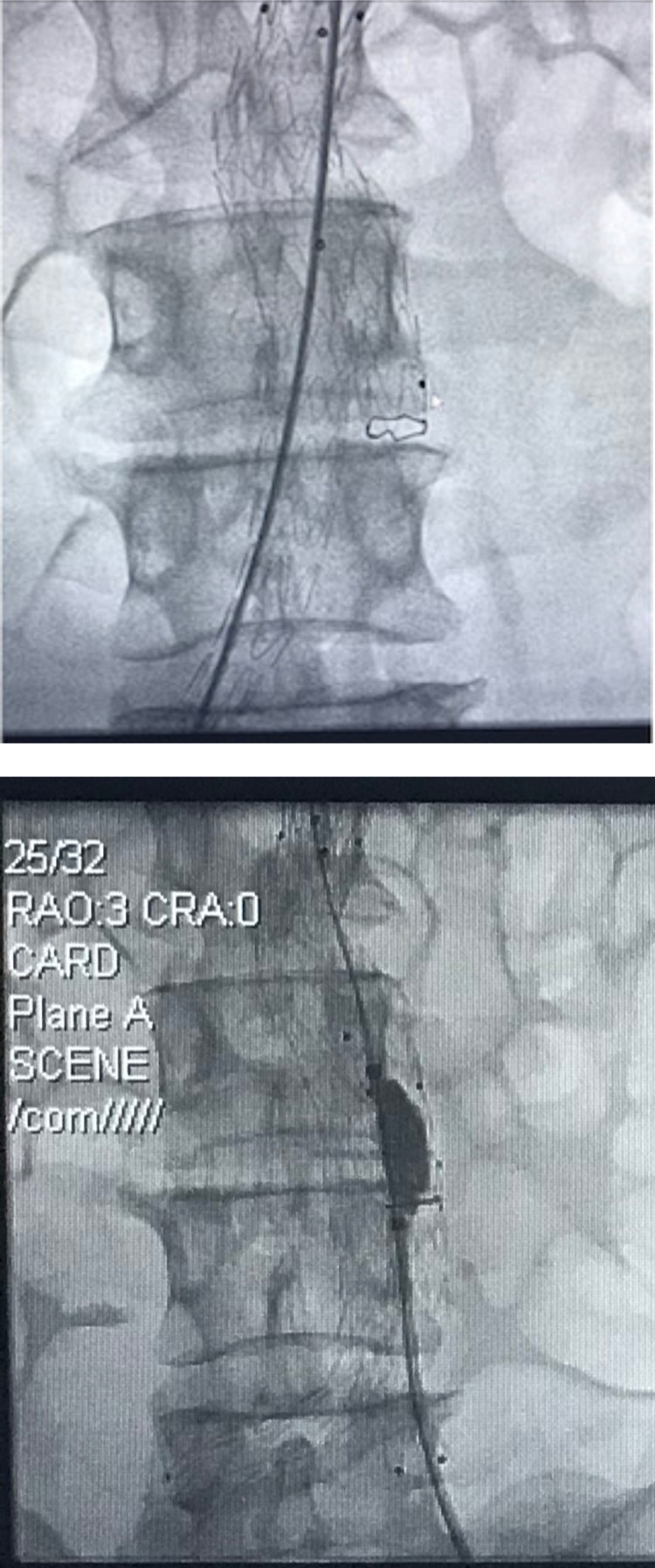
Deployment of bifurcate graft and subsequent molding with aortic balloon

The patient was admitted to hospital a day before the intervention. He was taken through normal pre-operative preparation, including signing consent and overnight fast. The intervention was done under local anaesthesia. He also had IV amoxiclav 1.2 g stat., IV paracetamol 1g stat. Access was via common femoral artery cut down bilaterally. Endurant bifurcate graft by Medtronic was deployed successfully under local anaesthesia (1% plain xylocaine with bupivacaine, 40 mls at a ratio of 50:50) and sedation with IV midazolam 15 mg. He also had IV paracetamol 1g stat at the start of intervention and then three times a day for three days, IV amoxiclav 1.2 g at the start of the procedure and twice a day for five days post-operative. Figures 2 and 3 show the intra-operative measurement of AAA and deployment of bifurcated graft and subsequent molding with the aortic balloon.

### Clinical Course

The post-operative period was uneventful. He started oral intake after 12 hours post-operative and mobilised independently from the first post-operative day. The wound drains were removed on day four post-operative and discharged from the hospital on postoperative day 5, making the total length of hospital stay six days. The patient had since been on surveillance with serial CT angiogram one-month post-discharge and subsequently once a year. 2021 is the 3^rd^ year of surveillance, and no complication has been detected.

Written informed consent for the case to be published (including images, case history and data) was obtained from the patient.

## Discussion

Data on AAA in Africa is scanty. Most of the papers published on the subject reported a prevalence of more than 3%.^8^–^10^ Most of the studies on AAA prevalence are from Northern Africa with a predominant Arab population.^10^,^11^ This h,ightens the need for more awareness and research on AAA in the Sub-Saharan region, including Ghana. Screening and prompt surgical treatment have been associated with a reduction in emergency surgery by 50–75%, rupture rate by 49–55% and mortality by 42–75%.^11^,^12^ Publishing surgical outcomes positively impact healthcare systems.^13^ Operative mortality associated with endovascular repair of abdominal aortic aneurysm is only a third of that associated with the open-repair procedure.^7^ Also, the length of hospital stay is shorter, 5–6 days, compared with open operative repair, where the median length of hospital stay is 11 days (interquartile range 9–14 days).^14^,^15^ Likelihood of blood transfusion is negligible and post-operative admission to the intensive care unit is not necessary. All these advantages make EVAR globally the current preferred treatment choice for AAA. The choice of EVAR for the treatment of AAA should be even more desirable in a healthcare resource-constrained country like Ghana. A reduction in length of hospital stay is crucial to free hospital bed space for the other numerous patients who require hospital admission. The availability of this novel vascular specialty skill, EVAR, is likely to improve the diagnosis and treatment outcome of patients with AAA in Ghana and positively impact the healthcare system.

We acknowledge that timely referral of the patient to the vascular clinic and subsequent prompt clinical evaluation and laboratory/ imaging workup culminated in successful EVAR. However, we observed that funding for the procedure was a major challenge since it was not covered by the National Health Insurance Scheme (NHIS). Lack of dedicated equipment and procedure materials in the country and a limited number of trained supporting staff on EVAR procedures were also major challenges that need to be addressed.

Within a reasonable period, these challenges notwithstanding, our hospital procured the basic equipment needed. Other supporting staff were also quickly mobilised into a team consisting of a vascular surgeon, assistant surgeons in training, operating theatre nurses, radiographers and an anaesthetist. These core team members were trained to assist with the case and all other Vascular cases after this index case. All variables considered, the total cost of EVAR in Ghana is a small fraction of what pertains in Europe and America. That said, the cost still falls outside the budget of most Ghanaians. Fortunately, our first patient had adequate financial support from family and friends to foot the bill.

A Vascular Specialist Clinic has been instituted at Korle Bu Teaching Hospital (KBTH). This allows the healthcare practitioners ample time outside the very busy General Surgery Clinics to evaluate and educate patients on their peculiar health conditions and other newer treatment modalities available in the country. There is also an additional day allocated for endovascular interventions. All these modifications culminated in the first successfully performed EVAR in the country. Further work in progress is to propose that the national health insurance scheme (NHIS) at least cover a significant fraction of the total cost of EVAR to make it affordable to a large number of patients. There have been presentations made in certain major hospitals in Accra over the period in terms of awareness creation. There are plans to expand this drive nationwide. The use of mainstream media is also an option being considered.

It is recommended that a dedicated centre for EVAR and other interventional procedures be set up for effective and efficient capacity building. Training of supporting staff for such procedures and the coverage of the cost of such procedures by the NHIS would help overcome some of the challenges faced currently.

## Conclusion

The definitive treatment for symptomatic AAA is surgical repair. Endovascular repair (EVAR) is associated with a much better peri-operative outcome than open repair. Our first case of EVAR is associated with no perioperative morbidity or mortality. EVAR is, therefore, a safe and effective alternative treatment for AAA.
